# Multichannel Two-Dimensional Convolutional Neural Network Based on Interactive Features and Group Strategy for Chinese Sentiment Analysis

**DOI:** 10.3390/s22030714

**Published:** 2022-01-18

**Authors:** Lin Wang, Zuqiang Meng

**Affiliations:** School of Computer and Electronic Information, Guangxi University, Nanning 530004, China; wanglingxun2021@163.com

**Keywords:** multichannel, two-dimensional convolutional neural network, interactive features, group strategy, feature mapping group

## Abstract

In Chinese sentiment analysis tasks, many existing methods tend to use recurrent neural networks (e.g., long short-term memory networks and gated recurrent units) and standard one-dimensional convolutional neural networks (1D-CNN) to extract features. This is because a recurrent neural network can deal with the order dependence of the data to a certain extent and the one-dimensional convolution can extract local features. Although these methods have good performance in sentiment analysis tasks, recurrent neural networks (RNNs) cannot be parallelized, resulting in time-inefficiency, and the standard 1D-CNN can only extract a single sample feature, with the result that the feature information cannot be fully utilized. To this end, in this paper, we propose a multichannel two-dimensional convolutional neural network based on interactive features and group strategy (MCNN-IFGS) for Chinese sentiment analysis. Firstly, we no longer use word encoding technology but use character-based integer encoding to retain more fine-grained information. Besides, in character-level vectors, the interactive features of different elements are introduced to improve the dimensionality of feature vectors and supplement semantic information so that the input matches the model network. In order to ensure that more sentiment features are learned, group strategies are used to form several feature mapping groups, so the learning object is converted from the traditional single sample to the learning of the feature mapping group, so as to achieve the purpose of learning more features. Finally, multichannel two-dimensional convolutional neural networks with different sizes of convolution kernels are used to extract sentiment features of different scales. The experimental results on the Chinese dataset show that our proposed method outperforms other baseline and state-of-the-art methods.

## 1. Introduction

Nowadays, social media and online shopping platform are widely used, and many users are happy to share their opinions and comments on social media and shopping platforms. Mastering and understanding these opinions and commenting on sentiment tendencies are essential to promote the healthy development of social media. To this end, there have been studies applying sentiment analysis to social media content, such as Twitter [1,2,3] and Weibo [4,5,6]. Some studies even predict political elections by analyzing the sentiment tendencies of content on social media. For example, political elections are predicted by analyzing relevant content on Twitter [7,8]. In any case, the reasonable use of sentiment analysis to automatically analyze a large number of content and comments on social media has great significance in the era of big data.

In the field of sentiment analysis, there are usually three methods based on a sentiment dictionary, based on traditional machine learning and based on deep learning. In early research, sentiment analysis methods based on a sentiment dictionary and sentiment analysis methods based on traditional machine learning are mostly used. With the development of deep learning, sentiment analysis based on deep learning has appeared in more and more sentiment analysis tasks.

The sentiment analysis method based on a sentiment dictionary often realizes the division of sentiment polarity at different granularities according to the sentiment polarity of sentiment words provided by different sentiment dictionaries. In terms of sentiment dictionaries, the construction of English-based sentiment dictionaries is relatively mature, and the earliest sentiment dictionary is SentiWordNet [9]. Other common ones include General Inquirer, Opinion Lexicon, and MPQA [10]. HowNet and NTUSD are frequently used in Chinese sentiment words. Asghar et al. [11] use domain terms, emoticons, negative words, and modifiers to enhance sentiment analysis to improve model performance. Han et al. [12] utilize SentiWordNet-based sentiment classifiers to score review datasets, and then select sentiment words from positive and negative reviews to train and generate domain sentiment dictionaries. Cai et al. [13] proposed the construction of a sentiment dictionary based on a specific domain in view of the polysemy problem in the sentiment dictionary. However, the sentiment dictionary-based method first needs to build a large-scale sentiment dictionary that contains as many sentiment words as possible, which is the basis for sentiment analysis by this method. However, the constructed sentiment dictionary cannot be updated automatically, which makes it very difficult to build a sentiment dictionary containing more sentiment words in reality when the word update speed is relatively fast. Besides, it takes a lot of time to construct a sentiment dictionary artificially, resulting in low time efficiency.

The sentiment analysis method based on traditional machine learning often trains a sentiment classifier through a given dataset, and then uses the sentiment classifier to predict sentiment polarity. Specifically, sentiment features are often first represented by statistical algorithms such as bag of words (BOW) [14], TF-IDF [15], N-grams [16], etc., and then the classifier is trained, and finally the classifier is used to predict sentiment polarity. In machine learning, common sentiment classifiers include support vector machine (SVM) [17], naive Bayes (NB) [18], and maximum entropy. In sentiment analysis tasks, decision trees (DT) and K-nearest neighbor (KNN) are sometimes also used as sentiment classifiers. Hajek [14] proposed to extract bag-of-words information and sentiment information from annual reports, and then combine the bag-of-words and sentiment features to predict stock investment reports. Dey et al. [15] aimed at the problem that existing methods only use TF-IDF to represent the unigram or n-gram feature vector; they proposed to use a combination of n-gram features and TF-IDF to represent sentiment features to improve the representation ability of sentiment features. Huq et al. [19] used KNN and SVM to classify the sentiment polarity of Twitter text. Dey et al. [16] used n-gram for feature extraction and added tags after the sentence, and then used the SVM classification algorithm for sentiment classification. Although some progress has been made in sentiment analysis based on traditional machine learning, due to the limitations of this method itself, it cannot represent sentiment features well, and the use of emotional information in the training process is limited.

In any case, the above two methods are mostly used in early sentiment analysis tasks and have poor feature representation capabilities. With the great success of deep learning in the image field, more and more researchers began to apply deep learning to the research of sentiment analysis and made great progress. Specifically, the common deep learning models used in the field of sentiment analysis are the convolutional neural network (CNN) [20,21,22], recurrent neural network (RNN) [23], long short-term memory network (LSTM) [24,25], gated recurrent unit (GRU) [26,27], etc. Among them, LSTM is a variant of RNN, and GRU is a variant of LSTM. Tang et al. [21] proposed graph convolutional networks for sentiment analysis, and Jelodar et al. [28] used LSTM to classify COVID-19 reviews. In fact, in order to further obtain sequence features, bidirectional long short-term memory network (BiLSTM) and bidirectional gated recurrent unit (BiGRU) are also often used in sentiment analysis; they learn sequence features from the front and back directions. Although the above models have made progress in sentiment analysis tasks compared to sentiment dictionary-based methods and traditional machine learning methods, a single neural network model still cannot fully extract sentiment features. For example, convolutional networks can only extract local features, while networks with sequence characteristics (e.g., LSTM, GRU, etc.) can only extract the entire sequence features. Therefore, a hybrid model combining convolutional networks and networks with sequence characteristics is proposed [29]. In addition, multichannel-based sentiment analysis methods have also been applied [27].

Based on the challenges of the above methods, and inspired by the great success of two-dimensional convolution in the image field and the multichannel network, we propose a multichannel two-dimensional convolutional neural network based on interactive features and group strategy (MCNN-IFGS) for Chinese sentiment analysis tasks. Specifically, our model is mainly composed of character-based integer encoding, interactive features, input tensor expansion based on group strategy, and multichannel two-dimensional convolutional neural networks with different convolution kernel sizes. The experimental results on the Chinese review dataset show that our method is effective. The main contributions of this paper are summarized as follows:

- The character-based integer encoding method is applied to the text sequence. Through this method, the feature extraction network can flexibly extract different levels of feature information, which is beneficial to retain more fine-grained information.
- The interactive features between character vector elements corresponding to any sample are introduced to improve the dimensionality of feature vectors and enhance sentiment features. We can obtain the corresponding semantic information by controlling the elements involved in the interaction.
- Group strategy has successfully realized the conversion of feature sequences into feature maps. Compared with traditional feature sequences, feature maps contain more feature information so that more sentiment features can be learned by the model.
- Multichannel two-dimensional convolutional neural networks with different convolution kernel sizes are used to extract sentiment features of different scales, which can effectively avoid the problem that single-channel networks cannot fully extract sentiment features.
- In the existing literature on sentiment analysis methods, we have implemented sentiment analysis based on two-dimensional convolutional neural networks for the first time and proposed a series of methods to ensure that two-dimensional convolutional neural networks are successfully applied to sentiment analysis tasks.

The rest of the paper is organized as follows. In Section 2, we put our approach in the context of relevant existing work. Then, in Section 3, we describe our method in detail, and the experimental evaluation and analysis are in Section 4. Section 5 is a summary.

## 2. Related Work

Sentiment analysis methods based on deep learning have become research hot spots, which are mainly divided into single network sentiment analysis and mixed network sentiment analysis. Among the sentiment analysis of a single network, sentiment analysis based on convolutional neural networks (CNNs) and long short-term memory networks (LSTM) have been used the most. Yin et al. [30] believe that it is difficult to fully extract sentiment features only by relying on end-to-end convolutional neural networks and the sentiment information of words can easily be ignored, so they proposed a sentiment lexical-augmented convolutional neural network (SCNN) for sentiment analysis. This method first learns the sentiment embedding from the sentiment dictionary, and then is input into the convolutional neural network as a text representation together with the word embedding for feature extraction and classification. Wang et al. [31] proposed a unified position-aware convolutional neural network (UP-CNN) to solve the problem that it is difficult to use important aspect position information when modeling aspect category sentiment analysis (ACSA) and aspect term sentiment analysis (ATSA) in a unified framework. This method first uses an aspect detection network with prior knowledge to solve the problem of the missing aspect position in ACSA, and then uses the aspect-aware context representation to fit the convolutional network.

Gan et al. [32] proposed a sparse attention-based separable dilated convolutional neural network (SA-SDCCN) for sentiment analysis to solve the problem of insufficient feature extraction by standard convolution, which can obtain features of different distances as much as possible without increasing parameters. Zhao et al. [33], Zhou et al. [34], and Lu et al. [35] successfully combined convolutional networks with graph models for sentiment analysis. Aiming at the problem that most existing methods in aspect-level sentiment classification ignore the sentiment dependence between different aspects, Zhao et al. [33] propose to use a graph convolutional network (GCN) to effectively capture the sentiment dependence information between different aspects in a sentence. Zhou et al. [34] proposed to use GCN to model syntax and knowledge for aspect-level sentiment analysis, thereby effectively improving the model’s use of syntactic information and common-sense knowledge. Aiming to solve the problem of ignoring syntactic constraints and long-range dependence in most existing methods in aspect-level sentiment analysis tasks, Lu et al. [35] proposed an aspect-gated graph convolutional network (AGGCN), which can effectively avoid the problem of erroneously identifying irrelevant context words as clues of emotion in judgment due to the above problems.

Sentiment analysis methods based on neural networks with sequence characteristics are also an important part of single network sentiment analysis. Common networks with sequence characteristics include LSTM, BiLSTM, GRU, and so on. Ma et al. [36] proposed using common-sense knowledge to solve the challenges faced by aspect-based sentiment analysis and targeted sentiment analysis. First, common sense related to sentiment concepts is incorporated into the training of the end-to-end network, and a Sentic LSTM is proposed to make common-sense knowledge better integrated in the recurrent encoder. Bao et al. [37] pointed out that the lack of flexibility of end-to-end deep neural networks makes it difficult to adjust the network to correct some obvious problems during the training process and the attention mechanism is unlikely to pay too much attention to the specific information of a sentence. Therefore, they proposed to use dictionary information to make the model more robust and flexible, and at the same time to use regularized attention to make the model pay more attention to different parts of the sentence.

Ahmed et al. [38] believe that traditional sentiment lexicons cannot avoid that the sentiment polarity of a word remains unchanged from one domain to another. Therefore, they proposed a weak supervised network model, which aims to learn a series of sentiment cluster embeddings from the global representation of sentences in the target domain, and then build a domain-dependent sentiment dictionary. Through this model, the problem of sentiment polarity change between domains can be well improved. Hassan et al. [39] proposed the use of gated-recurrent-units (GRUs) for a multi-class review sentiment classification task. This model combines word embedding in a specific domain that does not rely on reviewer information, which can help the model to learn sentiment features to a certain extent. Cambria et al. [40] have developed a three-level representation for sentiment analysis termed as SenticNet 5 which is able to discover conceptual primitives automatically, and the common-sense knowledge is embedded. After that, they proposed an ensemble of top-down and bottom-up learning embedded in senticNet 6, which is based in symbolic and subsymbolic AI [41]. They have trained their model using a WordNet-affect emoticon list, which is freely available on the internet. Of the above two methods, BiLSTM occupies a very important position. Wei et al. [42] proposed to use BiLSTM with multi-level orthogonal attention to perform implicit sentiment analysis tasks in response to the challenges of implicit sentiment analysis tasks. This method uses multi-level attention so that the difference between words and sentiment tendencies can be effectively identified as an important feature of implicit sentiment analysis. At the same time, the orthogonal mechanism is applied to ensure that the discriminant of the model is maintained during optimization.

Although the above sentiment analysis method based on a single network has made great progress, it cannot fully extract sentiment features. In order to obtain sentiment information more fully, sentiment analysis methods based on hybrid neural networks have been proposed one after another. Li et al. [43] and Behera et al. [44] successfully combined CNN and LSTM in sentiment analysis tasks. In response to this challenge, Li et al. [43] first proposed a new sentiment padding method based on integrated lexicon features, which can improve the proportion of sentiment information in each review, and secondly used a model based on the combination of CNN and LSTM to learn sentiment features. Behera et al. [44] proposed a sentiment analysis model called Co-LSTM based on CNN and LSTM for social big data. This model has strong adaptability and scalability in social big data, and secondly, it is not restricted by any specific domain. In addition, Zhang et al. [45] and Zhao et al. [46] successfully combined CNN and GRU for multimodal sentiment analysis tasks and aspect-based sentiment analysis tasks, respectively. Basiri et al. [47] proposed an ABCDM model that combines BiLSTM, BiGRU, and CNN for sentiment analysis tasks.

In addition, multichannel sentiment analysis methods are also an important part of hybrid neural network sentiment analysis. Gan et al. [48] pointed out that the existing sentiment analysis methods still face great challenges due to the serious multi-sentiment polarity of words and the long-term dependence between words in Chinese texts. Therefore, a scalable multichannel joint structure composed of CNN and BiLSTM with attention mechanism was proposed for sentiment analysis. The source context features and the multi-scale high-level context features can be extracted by the multichannel structure and the model channel can be adjusted according to the actual corpus. Feng et al. [49] proposed an MCNN-MA model to solve the problem of limited text features of short text, which is composed of a multichannel convolutional neural network with a multi-head attention mechanism. The model first combines word features with part-of-speech features, position information and dependency syntax features to form three new combined features, and then uses a convolutional neural network with a multi-head attention mechanism to further learn sentiment information. Aiming at the problem of easy loss of text information in short texts, Li et al. [50] further proposed a SAMF-BiLSTM model, which is composed of BiLSTM with a self-attention mechanism and multichannel features. This method first uses the existing language knowledge and sentiment resources to form different feature channels, and then uses the self-attention mechanism to enhance sentiment information. The model can make full use of the relationship between words and sentiment polarity without relying on sentiment dictionaries.

However, the abovementioned convolutional neural network is a one-dimensional convolutional neural network, which cannot fully extract sentiment features, and the neural network with sequential characteristics cannot be parallelized, which leads to low time efficiency. To this end, we propose a multichannel two-dimensional convolutional neural network (MCNN-IFGS) for sentiment analysis.

## 3. Our Method

**Figure 1:**
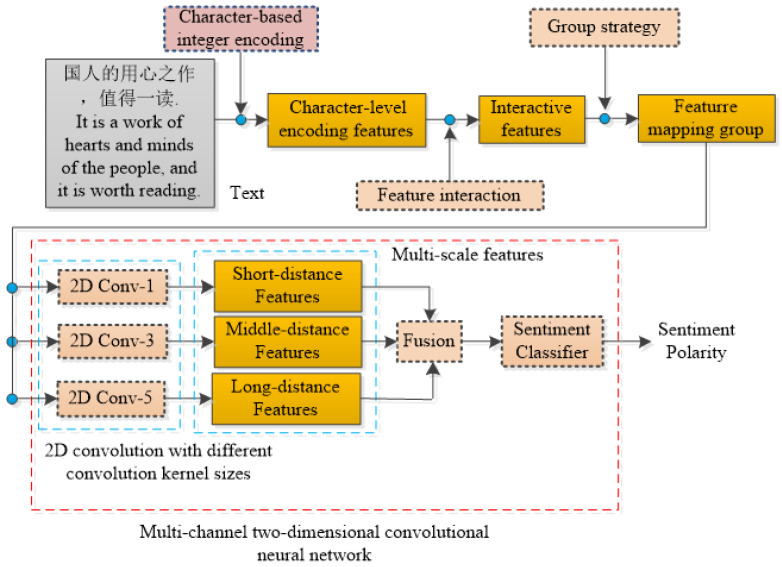
The framework of MCNN-IFGS. Here, 2D Conv-1 represents 2D-CNN with a size of 1*1 convolution kernel and so on.

Figure 1 illustrates the basic framework of our MCNN-IFGS method, which is mainly composed of four parts: a character-based integer encoding method, feature interaction, group strategy, and multichannel 2D-CNN with different convolution kernel sizes. First of all, the character-based integer encoding method is used to divide and encode text which can effectively retain more fine-grained information. Secondly, feature interaction method is used to generate interactive features to improve feature vector dimension and supplement semantic information. In addition, group strategies are used to form several feature mapping groups. Finally, multichannel 2D-CNN with different convolution kernel sizes is used to learn sentiment features at different scales.

### 3.1. Character-Level Encoding

Zhang et al. [51] pointed out that character-level information can provide flexible granular information. Inspired by this, character-based integer encoding is used to divide and encode text in this subsection. In Chinese, characters are the smallest unit of words, so through this encoding method we cannot only effectively retain semantic information but also retain fine-grained information.

Let $T=\{T_{1},T_{2},\ldots ,T_{i},T_{i+1},\ldots ,T_{n}\}$ denote the set of all text descriptions. For any text description $T_{i}$, we first use a character-based integer encoding method to divide the text, and the divided $T_{i}$ can be expressed as $T=\{T_{i,1},T_{i,2},\ldots ,T_{i,k},T_{i,k+1},\ldots ,T_{i,\alpha }\}$. Then, we encode the divided $T_{i}$ with integers to form the character-level vector, and the character-level vector after encoding can be expressed as $E=[E_{i,1},E_{i,2},\ldots ,E_{i,k},E_{i,k+1},\ldots ,E_{i,l}]$. Finally, all the character-level vectors corresponding to the text description are merged into a character-level matrix E, which can be expressed as:

(1) $$\begin{array}{ll} E & =Merge\left(E_{1},E_{2},\ldots ,E_{i},E_{i+1},\ldots ,E_{n}\right)\\ & =\left[E_{1},E_{2},\ldots ,E_{i},E_{i+1},\ldots ,E_{n}\right] \end{array}$$

where α represents the character length of the text description, l represents the dimension of the character-level vector, and Merge( ) a acts as a merger, $E_{i}\in {\mathbb{R}}^{l},E\in {\mathbb{R}}^{n\times l}$.

### 3.2. Interactive Features

As described in the previous subsection, text-based character-level vectors alone do not provide enough semantic information. Therefore, in this subsection, interactive features are introduced to supplement semantic information to make up for the deficiency of character-level vectors to achieve the purpose of enhancing sentiment. In addition, through interactive features, we have successfully increased the dimension of character-level vectors, paving the way for the smooth introduction of group policies in the next subsection.

#### 3.2.1. Definition of Interaction Features

The interaction features are defined as follows:

In the character-level vector $E_{i}$, the element $e_{i,p}$ corresponding to the position p(p=0,1,2…,l) is sequentially multiplied with the $\beta (\beta \in N_{+})$ elements corresponding to the remaining positions until the multiplication operation cannot be performed. In this process, we call the multiplication operation an interactive behavior, and the element generated by each multiplication is called an interactive feature, and all the interactive features generated by the element $e_{i,p}$ together constitute the interactive feature vector $M_{p}$ of the element $e_{i,p}$. Finally, all $M_{p}$ are concatenated into interactive feature vectors $F_{i,\beta }$ corresponding to character-level vectors $E_{i}$.

It is particularly important to note that the elements at the same position do not participate in interactive calculations, and the β elements participating in interactive calculation each time are directional, and the corresponding positions are arranged from low to high.

#### 3.2.2. Representation of Interactive Feature Vectors

First of all, in order to better express the interactive feature vectors, we have introduced some special symbols, which are explained as follows:

- U represents the vector continuous concatenation symbol. Assuming $M_{1}=[1,2,3,4],M_{2}=[5,6,7,8]$, then:
  (2) $$\begin{array}{ll} \overset{2}{\underset{p=1}{U}}M_{p} & =[M_{1},M_{2}]\\ & =[1,2,3,4,5,6,7,8] \end{array}$$
- C represents the combining marker in mathematics
- Concatenate() has the same effect as U
- fuse() means to fuse two vectors into a two-dimensional matrix, for example:
  (3) $$\begin{array}{ll} F & =fuse\left(\;M_{1},M_{2}\right)\\ & =[M_{1},M_{2}]\\ & =[[1,2,3,4],[5,6,7,8]] \end{array}$$

Then, the interactive feature vector $F_{i,\beta }$ can be expressed as:

(4) $$w=\overset{p}{\underset{\gamma =1}{\sum }}C_{l-\gamma }^{\beta -1}$$

(5) $$Q=F_{i,\beta -1\;\;}^{w}$$

(6) $$M_{p}=e_{1,p}\cdot Q$$

(7) $$\begin{array}{ll} F_{i,\beta } & =\overset{l-\beta }{\underset{p=1}{U}}e_{1,p}\cdot Q\\ & =\overset{l-\beta }{\underset{p=1}{U}}M_{p}\\ & =[M_{1},M_{2},\ldots ,M_{p},M_{p+1},\ldots ,M_{l-\beta +1}] \end{array}$$

In the above expression, the meaning of each symbol is explained as follows:

- w represents the first w term of the interactive feature vector $F_{i,\beta }$
- Q represents the interactive feature vector after removing the first w interactive features in $F_{i,\beta }$
- $M_{p}$ represents the interactive feature vector generated by element $e_{i,p}$
- where $Q\in {\mathbb{R}}^{C_{l}^{\beta -1}-\omega }$, $M_{p}\in {\mathbb{R}}^{C_{l}^{\beta -1}-\omega }$, $F_{i,\beta }\in {\mathbb{R}}^{C_{l}^{\beta +1}}$, the initial interactive feature vector $F_{i}^{0}=E_{i}$.

Next, the interactive feature vector $F_{i,\beta }$ corresponding to different β and the initial interaction feature vector f are concatenated into a new interactive feature vector $F_{i}$, which can be expressed as:

(8) $$\begin{array}{ll} F_{i} & =Concatenate\left(F_{i,0},\;F_{i,1},F_{i,2},\ldots ,F_{i,\beta },\ldots ,F_{i,m}\right)\\ & =\overset{m}{\underset{\beta =1}{U}}F_{i,\beta }\\ & =[F_{i,0},\;F_{i,1},F_{i,2},\ldots ,F_{i,\beta },\ldots ,F_{i,m}] \end{array}$$

where $F_{i}\in {\mathbb{R}}^{d_{i}},d_{i}=\overset{m}{\underset{\beta =1}{\sum }}C_{l}^{\beta }+l$.

Finally, different interaction feature vectors $F_{i}$ are fused into a two-dimensional interaction feature vector F.

(9) $$\begin{array}{ll} F & =fuse\left(\;F_{1},F_{2},\ldots ,F_{i},F_{i+1},\ldots ,F_{n}\right)\\ & =[F_{1},F_{2},\ldots ,F_{i},F_{i+1},\ldots ,F_{n}] \end{array}$$

where $F\in {\mathbb{R}}^{n\times d},d=\overset{m}{\underset{\beta =1}{\sum }}C_{l}^{\beta }+l$. The function of fuse( ) is to fuse the different $F_{i}$ into a two-dimensional interaction feature vector.

#### 3.2.3. The Determination Principle of β

From expressions Equations (3)–(6), we can know that when the length is fixed, β determines the number of interactive features generated; that is, the dimension of the interactive feature vector. Therefore, choosing the appropriate β is conducive to supplementing enough semantic information to achieve the effect of sentiment enhancement. Secondly, choosing a reasonable β helps to reduce the dimensionality of the interactive feature vector, improve the generation efficiency, and avoid the dimensional disaster and time consumption caused by a too-large β value. In order to select the appropriate β more accurately, we count the number distribution of words corresponding to different word lengths from the Chinese review dataset. According to the distribution, we choose the length with more words as the value range of β. According to statistics, we find that the words of the dataset are mainly concentrated in lengths 1, 2, and 3 and the proportion of the number of words corresponding to these three lengths is more than 90%. Therefore, in this paper, we take 1 and 2 as the value of β. It is specially noted that when the word length is 1, the corresponding a = 0 means that no interaction will be performed, so 0 is not used as the value of β.

For the interactive feature matrix F, PCA is used to reduce the vector dimension. Assuming that the specified dimension is b, the new interactive feature matrix $F^{\prime }$ after PCA dimensionality reduction satisfies $F^{\prime }\in {\mathbb{R}}^{n\times b}$.

### 3.3. Group Strategy

In sentiment analysis tasks, the standard one-dimensional convolutional neural network often cannot learn enough sentiment features. There are two main reasons: one is that the standard one-dimensional convolutional neural network can only extract local features, and the other is the learning object of the model is often a single sample in sentiment analysis tasks. In this subsection, we mainly discuss how to use group strategy to solve the above problems.

In order to solve the above problems, our idea is to convert the traditional two-dimensional feature matrix into several feature mapping groups as model input. However, a simple way to switch between the two cannot guarantee that the emotional polarity of the transformed feature map group remains unchanged. To this end, we propose to use group strategy to perform this conversion process. This method first divides the samples with the same sentiment polarity into several groups, so the samples of the same group have the same sentiment polarity. Then, we convert the samples in each group to ensure that the sentiment polarity of the converted feature map group remains unchanged. Finally, each feature mapping group is converted from a number of samples with the same sentiment polarity. Through the group strategy, we successfully converted the learning object of the model from a single sample to a feature mapping group, which is conducive to learning more sentiment features.

Specifically, for the interactive feature matrix $F^{\prime }$, we divide the samples into different groups according to the sentiment polarity $S_{k}$. Here, we use $B^{S_{k}}\in {\mathbb{R}}^{n_{S_{k}}\times b}$ to represent the sample set corresponding to sentiment polarity $S_{k}$, and $n_{S_{k}}$ to represent the number of samples corresponding to sentiment polarity $S_{k}$. In $B^{S_{k}}$, all samples have the same sentiment polarity.

It is worth noting that in the conversion process, the dimension corresponding to each dimension has some influence on the model performance, so choosing the appropriate dimension for each dimension has a positive impact on improving the model performance. Encouraged by the representation of image features, in the conversion process, we keep the dimensions of the second dimension and the third dimension of the input tensor equal. In order to make the model learn as much sentiment information as possible, the fourth dimension is determined according to the principle of taking a larger value for a large sample and a smaller value for a small sample. Specifically, assuming J is the dimension of the fourth dimension, then $B^{S_{k}}$ is first converted into $D^{S_{k}}$ feature mapping groups, each feature mapping group consists of J samples, and J satisfies $J=\frac{n_{S_{k}}}{D^{S_{k}}}$. In this paper, because we use a large-scale review dataset, according to the above principles, we choose a series of larger J values to conduct experiments to determine the most appropriate J value.

Finally, $B^{S_{k}}$ is converted into the four-dimensional input tensor $B^{S_{k}}\in {\mathbb{R}}^{D^{S_{k}}\times H\times I\times J}$. All the converted input tensors $B^{S_{k}}$ are finally fused into a new four-dimensional input tensor $B^{S}\in {\mathbb{R}}^{D^{S}\times H\times I\times J}$, which can be expressed as:

(10) $$\begin{array}{ll} B^{S} & =fuse\left(\;B^{S_{1}},B^{S_{2}},\ldots ,B^{S_{k}},B^{S_{k+1}},\ldots ,B^{S_{h}}\right)\\ & =[B^{S_{1}},B^{S_{2}},\ldots ,B^{S_{k}},B^{S_{k+1}},\ldots ,B^{S_{h}}] \end{array}$$

where $H=I=\sqrt{b}$, $D^{S}=D^{S_{1}}+\ldots +D^{S_{h}}$. For the extended four-dimensional input tensor $B^{S}$, it can be regarded as consisting of $D^{S}$ feature mapping groups $M\in {\mathbb{R}}^{H\times I\times J}$. Each feature mapping group M consists of J samples with the same sentiment polarity.

### 3.4. Multichannel Two-Dimensional Convolutional Neural Networks

**Figure 2:**
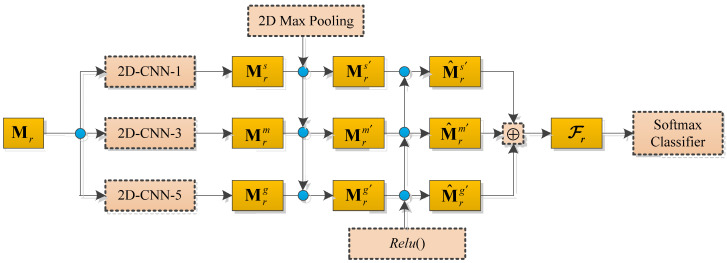
Multichannel two-dimensional convolutional neural network.

In this subsection, a multichannel two-dimensional convolutional neural network is used for sentiment analysis, and each two-dimensional convolution has a different convolution kernel size, which is conducive to learning sentiment features of different scales. In addition, compared to a single-channel network, a multichannel network can obtain more sentiment information. Figure 2 shows the main structure of the multichannel two-dimensional convolutional neural network.

In this paper, we propose to utilize two-dimensional convolution with different convolution kernel sizes to extract sentiment features of different scales. In order to ensure that the second dimension and the third dimension of the input and output feature tensors before and after convolution remain unchanged, before convolution, any feature mapping group $M_{r}$ is 0-padded. Specifically, we utilize two-dimensional convolutions with 1 × 1, 3 × 3, and 5 × 5 convolution kernel sizes to extract short-distance, middle-distance, and long-distance sentiment features, respectively. After that, two-dimensional max-pooling was used to select useful sentiment features. This process can be expressed as:

(11) $$M_{r}^{s}=tanh\left(\;M_{r}*W_{s}\right)$$

(12) $$M_{r}^{s^{\prime }}=max\left(\;M_{r}^{s}\right)$$

(13) $$M_{r}^{m}=tanh\left(\;M_{r}*W_{m}\right)$$

(14) $$M_{r}^{m^{\prime }}=max\left(\;M_{r}^{m}\right)$$

(15) $$M_{r}^{g}=tanh\left(\;M_{r}*W_{g}\right)$$

(16) $$M_{r}^{g^{\prime }}=max\left(M_{r}^{g}\right)$$

where $M_{r}^{s}\in {\mathbb{R}}^{H\times I\times J_{s}}$, $M_{r}^{m}\in {\mathbb{R}}^{H\times I\times J_{m}}$ and $M_{r}^{g}\in {\mathbb{R}}^{H\times I\times J_{g}}$ are two-dimensional convolution outputs with 1 × 1, 3 × 3, and 5 × 5 convolution kernel sizes, respectively $J_{s}$, $J_{m}$ and $J_{g}$ are the dimensions of the corresponding two-dimensional convolution output space $M_{r}^{s^{\prime }}\in {\mathbb{R}}^{H_{s}\times I_{s}\times J_{s}}$, $M_{r}^{m^{\prime }}\in {\mathbb{R}}^{H_{m}\times I_{m}\times J_{m}}$ and $M_{r}^{g^{\prime }}\in {\mathbb{R}}^{H_{g}\times I_{g}\times J_{g}}$ are the corresponding two-dimensional max-pooling outputs. $H_{s}=I_{s}=H_{m}=I_{m}=H_{g}=I_{g}=\frac{H}{2}=\frac{I}{2}$ and $W_{s}$, $W_{m}$ and $W_{g}$ are the corresponding two-dimensional convolution weight parameters, respectively. tanh is the activation function of the two-dimensional convolution.

For feature matrices $M_{r}^{s^{\prime }}$, $M_{r}^{m^{\prime }}$ and $M_{r}^{g^{\prime }}$, they are respectively embedded in a V-dimensional output space for feature fusion in the later stage. After embedding, all output feature matrices have the same shape. This embedding can be expressed as:

(17) $${\hat{M}}_{r}^{s^{\prime }}=relu\left(W^{s^{\prime }}M_{r}^{s^{\prime }}+b_{s^{\prime }}\right)$$

(18) $${\hat{M}}_{r}^{m^{\prime }}=relu\left(W^{m^{\prime }}M_{r}^{m^{\prime }}+b_{m^{\prime }}\right)$$

(19) $${\hat{M}}_{r}^{g^{\prime }}=relu\left(W^{g^{\prime }}M_{r}^{g^{\prime }}+b_{g^{\prime }}\right)$$

where ${\hat{M}}_{r}^{s^{\prime }}\in {\mathbb{R}}^{H_{s}\times I_{s}\times V}$, ${\hat{M}}_{r}^{m^{\prime }}\in {\mathbb{R}}^{H_{m}\times I_{m}\times V}$ and ${\hat{M}}_{r}^{g^{\prime }}\in {\mathbb{R}}^{H_{g}\times I_{g}\times V}$.$W^{s^{\prime }}$,$W^{m^{\prime }}$,$W^{g^{\prime }}$, and $b_{s^{\prime }}$, $b_{m^{\prime }}$, $b_{g^{\prime }}$ are parameters, relu is the activation function of the fully connected layer. After that, the element-wise addition method is used to fuse ${\hat{M}}_{r}^{s^{\prime }}$, ${\hat{M}}_{r}^{m^{\prime }}$ and ${\hat{M}}_{r}^{g^{\prime }}$ to obtain the fusion feature ${ℱ}_{r}$, which can be expressed as:

(20) $${ℱ}_{r}={\hat{M}}_{r}^{s^{\prime }}⊕{\hat{M}}_{r}^{m^{\prime }}⊕{\hat{M}}_{r}^{g^{\prime }}$$

where ⊕ is the element-wise addition.

Finally, the fusion feature ${ℱ}_{r}$ is input into a softmax classifier for sentiment classification, and cross-entropy is used as the loss function of the model. During the training process, the model parameters are optimized by minimizing cross-entropy

(21) $${\hat{y}}_{i}=softmax\left(W_{y}{ℱ}_{i}\right)$$

(22) $$ℒ=-\frac{1}{N}\sum_{i}\sum_{j}y_{i}\log p({\hat{y}}_{i})$$

where $W_{y}$ is the parameters of the softmax function, i is the index of the text description, j is the category index, ${\hat{y}}_{i}$ denotes the predicted value, and $y_{i}$ is the ground truth.

## 4. Experiments

In this section, extensive experiments are outlined that verify the effectiveness of MCNN-IFGS. Specifically, the experimental setups are first stated. Secondly, comparative experiments are detailed, and the results of the experiments are analyzed. Finally, we show the additional experiments conducted to further verify the effectiveness of our method.

### 4.1. Experiment Setups

#### 4.1.1. Datasets

**Figure 3:**
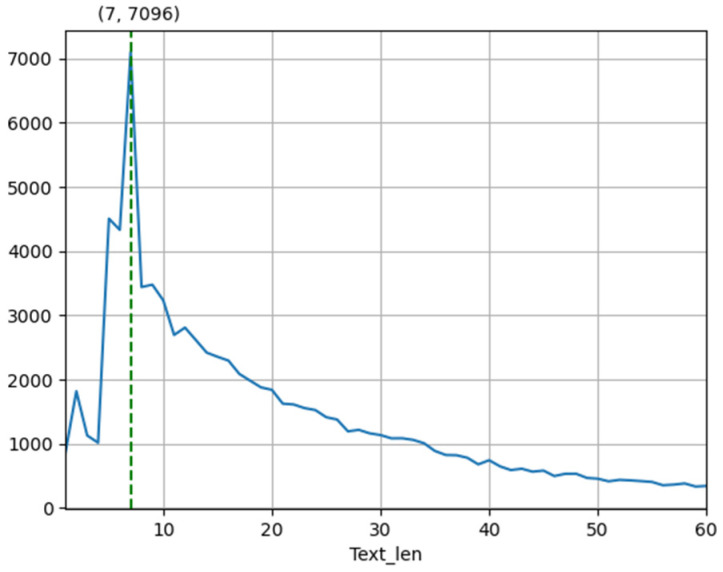
The changes in the number of samples corresponding to different text lengths.

**Figure 4:**
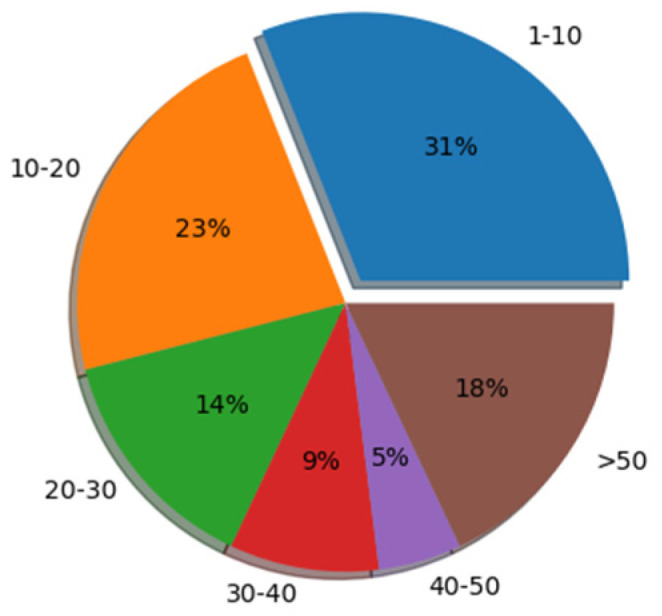
The probability distribution corresponding to different text length ranges.

The experiment was carried out on a dataset of Chinese reviews from popular shopping websites in China [52]. The dataset included 100,000 shopping reviews, and each review corresponded to different sentiment polarity, positive or negative. In this paper, in order to maintain category balance, we randomly selected 97,200 reviews from the dataset, and used the standard train/test splits of 72,900/24,300 for the sentiment analysis task. The statistics of the dataset are shown in Table 1. In order to obtain more text information of the dataset, the text sequence length distribution of the dataset was counted as shown in Table 2, and the changes in the number of samples corresponding to different text lengths are shown in Figure 3. Figure 4 shows the probability distribution corresponding to different text length ranges.

#### 4.1.2. Data Preprocessing

In the original dataset, there were illegal characters due to encoding problems, which affected the performance of the model to a certain extent. In order to avoid the impact of illegal characters on the performance of the model as much as possible, we corrected the encoding problem and removed illegal characters. In this paper, in order to protect the integrity of the text sequence, stop words and punctuation have not been removed. Finally, the preprocessed text and the corresponding sentiment polarity were written into a text file as the experimental operation object, and at the same time facilitated the statistics of the text data distribution. 

#### 4.1.3. Evaluation Metrics

In our research, in order to evaluate the performance of the model more comprehensively, we used Accuracy, Precision, Recall, and F1-score as evaluation metrics, which are widely used in sentiment analysis tasks. The standard deviation of each evaluation metric score was used to measure the stability of each evaluation metric. In addition, in order to avoid accidental errors as much as possible to ensure the validity of the experimental results, each method was run five times in the experiment, and the average of the different evaluation metrics was used as the final result to quantify.

#### 4.1.4. Hyperparameter Setting

In this work, the most appropriate value of input channel J was set to 60 and the value of the compressed dimension b of PCA was set to 900. Thirty-two 1 × 1 filters, sixty-four 3 × 3 filters and one hundred and twenty-eight 5 × 5 filters were respectively set as the first two-dimensional convolutional layer, the second two-dimensional convolutional layer, and the third two-dimensional convolutional layer parameters. In order to better train the optimal model, we chose Adam as the optimization algorithm to train our network, and the optimal learning rate in this optimization algorithm was set to 0.001 for this sentiment analysis task. We set the number of training epoch to 30, the batch size to 128, and the dropout rate to 0.8.

### 4.2. Comparison with Existing Methods

#### 4.2.1. Comparative Methods

- *LSTM:* In this method, a LSTM network is used to extract sentiment features. It is composed of a embedding layer, an LSTM layer and a full connected layer [53].
- *Two-layer LSTM:* A two-layer LSTM is used to extract text features [54].
- *BiLSTM:* Bidirectional long short-term memory network. A one-layer BiLSTM is used to extract text features [54].
- *Two-layer BiLSTM:* A two-layer BiLSTM is used to extract text features [54].
- *GRU:* A gated recurrent unit is a variant of LSTM. Compared with LSTM, GRU retains its resistance to gradient disappearance. Meanwhile, its internal structure is simpler, training is faster, and it has been widely used for sentiment analysis recently [55].
- *BiGRU:* Bidirectional gated recurrent unit. In order to solve the difficult problem of modeling sentiment relationships in recurrent structure, Chen et al. [56] proposed to use a bidirectional gated recurrent unit to capture sentimental relationship information.
- *Character-level ConvNets:* This method applies ConvNets to characters for the first time. Experimental results show that when training on large-scale datasets, ConvNets do not require word-level information. In addition, existing research results show that ConvNets do not rely on semantic information [57].
- *SLCABG:* This method uses a convolutional neural network (CNN) combined with a bidirectional gated recurrent unit (BiGRU) for sentiment analysis of Chinese text. The attention mechanism and the sentiment lexicon are used to emphasize important information and enhance sentiment features, respectively [58].
- *MDMLSM:* This model firstly uses the pre-trained BERT model to form word vectors, then applies the attention-based BiLSTM to extract text features, and finally the output feature representations are sequentially input into the multilayer perceptron and sentiment classifier [59].
- *MC-2D-CNN (word-based):* In this article, the MCNN-IFGS we propose is based on character-based operation objects. In order to further explore the differences between characters and words, we added the word-based MCNN-IFGS as a comparison method.

Here, the stochastic gradient descent (SGD) is set to the optimization algorithm of character-level ConvNets, GRU, and BiGRU to train the network, where the learning rate is 0.01. LSTM, two-layer LSTM, BiLSTM, and two-layer BiLSTM use Adagrad as an optimization algorithm to train the model, and the learning rate is 0.05. Adam is used as the optimization algorithm of MCNN-IFGS (word-based), and the learning rate is set to 0.001. It is worth noting that all the above parameters were set according to the original paper and appropriately adjusted according to the Chinese dataset to maintain the best state of the model.

#### 4.2.2. Results and Analysis

A series of comparative experiments were carried out on the Chinese review dataset. The experimental results are shown in Table 3.

From Table 3, we can know that our MCNN-IFGS method is superior to six recurrent neural network methods. Among the six methods, GRU has the worst model performance, and its accuracy, precision, recall, and F1-score scores reached only 86.7%, 87.1%, 86.4%, and 86.8%, respectively, and the scores of the other five methods were similar. The overall performance of the two-layer LSTM method was the best, and the accuracy score reached 93.5%, which was 0.3%, 0.4%, 0.5%, 6.8%, and 0.5% higher than that of the other five methods. From the value of the standard deviation, the scores of each evaluation metric of these five methods were relatively stable, and the degree of fluctuation was small. The maximum amplitude of fluctuation appeared in the recall of GRU, and the value of standard deviation was 0.006. In any case, the scores of our MCNN-IFGS method on the four evaluation metrics were all higher than those of the six recurrent neural network methods, and the scores on accuracy, precision, recall, and the F1-score were higher than 3.7%, 4.2%, 4.2%, and 4.2% of the scores of the four evaluation metrics of two-layer LSTM, respectively. This shows that compared with the six recurrent neural network methods, our MCNN-IFGS method can learn more sentiment features, all thanks to our conversion of the learning object from a traditional single sample to feature mapping group.

We can also see from Table 3 that our MCNN-IFGS method was superior to character-level ConvNets, SLCABG, and MDMLSM. In these three methods, the model performance of SLCABG was better than that of character-level ConvNets. The accuracy, precision, recall, and F1-score scores of SLCABG were 93.4%, 93.1%, 93.7%, and 93.4%, respectively, which were 0.6%, 0.2%, 0.7%, and 0.5% higher than those of character-level ConvNets. This is because the SLCABG method uses 1D-CNN and BiGRU to extract features and the attention mechanism is used to focus on important features. Compared with the standard 1D-CNN, it can obtain and utilize relatively more features. In fact, from the score of the evaluation metrics, the score gap between character-level ConvNets and SLCABG was not large. However, CNN can be parallelized in the training process with high time efficiency, while a recurrent neural network cannot be parallelized, resulting in a high time cost. From the perspective of time cost, the use of the CNN method for sentiment analysis tasks has certain advantages. Besides, we observe that the model performance of MDMLSM was slightly higher than that of character-level ConvNets, which is because MDMLSM uses an attention-based BiLSTM network to help the model capture important information. In any case, the above three methods were weaker than our MCNN-IFGS method. The scores of our MCNN-IFGS method on the four evaluation metrics were 3.8%, 4.3%, 3.8%, and 4.0% higher than those of SLCABG, respectively, indicating that our method was more effective.

From Table 3, we can see that our MCNN-IFGS method was relatively superior to MCNN-IFGS (word-based), and its scores on accuracy, recall, and F1-score were higher than 0.2%, 0.9%, and 0.2% of the corresponding evaluation metrics of MCNN-IFGS (word-based), respectively. This shows that the character-based method of constructing objects can be more helpful to improve the performance of the model. Compared with the word-based method of constructing objects, it can provide or help us retain more sentiment information.

### 4.3. Further Analysis of MCNN-IFGS

In this section, in order to further prove the effectiveness of our MCNN-IFGS method, we show the design of two further experiments.

#### 4.3.1. Effect of Learning Rate

**Figure 5:**
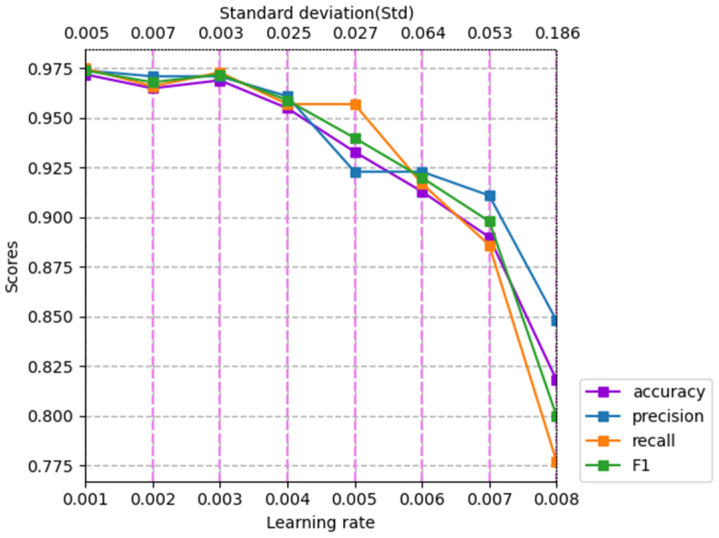
Model performance comparison of different learning rates on the Chinese review dataset. The value at the top of the violet dotted line represents the average of the standard deviations of all evaluation metrics scores corresponding to different dropout values.

In this subsection, in order to explore the influence of the learning rate of the optimization algorithm on the MCNN-IFGS method, we conducted a series of experiments for different learning rates. In particular, we set a series of learning rates to {0.001, 0.002, 0.003, 0.004, 0.005, 0.006, 0.007, 0.008}. The experimental results are shown in Table 4 and Figure 5.

From Table 4 and Figure 5, we can conclude that when the learning rate was 0.001, the model had the best performance, and its scores on accuracy, precision, recall, and F1-score reached 97.2%, 97.4%, 97.5%, and 97.4%, respectively. On the whole, the performance of the model decreased as the value of the learning rate increased. When the value of the learning rate was 0.008, the scores on accuracy, precision, recall, and F1-score decreased to 81.8%, 84.8%, 77.7%, and 80.0%, respectively, and the model performance was the worst. Specifically, from the perspective of different intervals, as the value of the learning rate increased from 0.001 to 0.003, the model performance showed a trend of first decline and then rise, and the decline of the four evaluation indicators reached 0.7%, 0.2%, 1.1%, and 0.5%, respectively. Although there was a fluctuation in the model performance between 0.001 and 0.003, their fluctuation amplitude is relatively small, indicating that the learning rate in the interval had limited influence on the model performance and the model performance was relatively stable. As the learning rate exceeded 0.003, the performance of the model showed a stable downward trend. When the learning rate increased to 0.008, the score of each evaluation metric of the model was the worst, indicating that with the learning rate exceeding 0.003, the negative impact of the learning rate on the model began to appear, and increased with the increase of the learning rate value. These negative impacts led to the decline of the model’s ability to extract and utilize sentiment features, which further affected the performance of the model.

From the perspective of model stability, the stability of model performance was the worst when the learning rate was 0.008, and the average of the standard deviation of all evaluation metric scores was 0.186. In contrast, the stability of the model’s performance was the best when the learning rate was 0.003, and the average of the standard deviation of all evaluation metric scores was 0.003. From the overall trend of change, the average of the standard deviation of all evaluation metric scores increased with the increase of learning rate, which indicates that the stability of the model performance decreased with the increase of learning rate, and this instability would also affect the ability of the model to extract and utilize sentiment features. In any case, we can also see from Figure 5 that when the learning rate was in the range of 0.001 to 0.003, the stability of the model had a trend of first rise and then decline; the average of the standard deviation increased from 0.005 to 0007 and then decreased to 0.003, but the maximum volatility was only 0.007 and the average of the standard deviation was only 0.007, far lower than the value of the rest of the interval. This shows that although there were fluctuations in this interval, the stability of the model had little effect, and the stability of the model was relatively good. As the learning rate exceeded 0.003, this fluctuation disappeared, accompanied by a stable upward trend, indicating that the stability of the model changed significantly when the learning rate exceeded 0.003, and the unstable factors increased, which is not conducive to the model fully learning sentiment features.

In short, through the above analysis, we can know that the learning rate has a significant impact on the performance and stability of the model. Choosing the appropriate value of the learning rate plays an extremely important role in improving the performance of the model and maintaining the stability of the model. In fact, the main reason why different learning rates affect the model is that an inappropriate learning rate will make the model unable to fully learn the text features during the training process.

#### 4.3.2. Effect of Model Parameters

**Figure 6:**
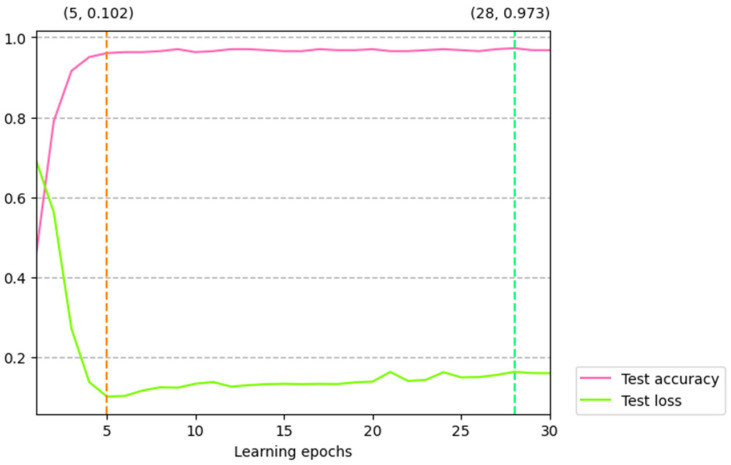
The test accuracy and test loss value change with the learning epochs. The value at the top of the spring green dashed line represents the learning epoch with the maximum test accuracy and its corresponding accuracy, and the value at the top of the lawn green dashed line represents the learning epoch with the minimum test loss and its corresponding test loss value.

In this subsection, we detail how in previous work, model parameters often had a certain degree of influence on the model. Here we mainly explore the specific impact of dropout value and learning epoch on the MCNN-IFGS method. Specifically, in the experiment to explore the influence of dropout value on the model, we set the dropout value as {0.2, 0.3, 0.4, 0.5, 0.6, 0.7, 0.8}, and the experimental results on different dropout values are shown in Table 5. In the experiment to explore the impact of learning epoch on the model, the maximum value of the learning epochs was set to 30, and the test accuracy and test loss were used as evaluation metrics. Figure 6 shows the changes of test accuracy and test loss value with learning epochs.

From Table 5, we can see that the overall performance of the model was basically better and better as the dropout value increased. Although there were some fluctuations, these fluctuations were small and not enough to affect the overall change law. As the dropout value increased to 0.8, the model learned best. In any case, as the dropout value increased to 0.3, the model performed the worst. Its scores on accuracy, precision, recall, and F1-score only reached 96.2%, 96.7%, 96.5%, and 96.6%, respectively. Specifically, we found the difference in model performance corresponding to different dropout values was not large, which shows that different dropout values had less impact on the model performance. From the perspective of model stability, model performance was basically stable. The average value of the standard deviation of all evaluation metrics scores corresponding to different dropout values was relatively small, and the maximum value was only 0.007. In short, the above analysis found different dropout values had less negative effects on model performance and model stability.

From Figure 6, we can observe that the change curve of test accuracy with learning epochs was relatively smooth, and the change of test accuracy relatively stable. Specifically, we found that when learning epochs were between 1 and 5, the test accuracy increased with the increase of learning epochs. As the number of learning epochs exceeded 5, the accuracy of the model no longer changed greatly but began to maintain a relatively stable state. When the number of learning epochs increased to 28, the accuracy of the model reached the maximum. This change shows that when the learning round was 1 to 5, the learning ability of the model increased rapidly. As the learning round exceeded 5, this learning ability of the model did not change significantly, and the model’s learning of sentiment features reached a certain height.

We can also see that the change in the test loss value was basically the opposite of the change in the test accuracy from Figure 6. The test loss value decreased sharply with the increase of the learning rounds when the learning epochs were between 1 and 5. This shows that the learning ability of the model increased rapidly in this interval. As the learning epoch increased to 5, the test loss value reached the minimum value of 0.102. As the learning epoch exceeded 5, the test loss value no longer changed significantly but was in a state of minimal fluctuation.

## 5. Conclusions

In order to improve the problem that traditional sentiment analysis methods cannot fully learn Chinese sentiment features, we proposed a multichannel two-dimensional convolutional neural network based on interactive features and group strategy (MCNN-IFGS). The core idea of the paper was to convert the learning object of the model from a single sample to a feature mapping group, and then use MCNN-IFGS for emotional feature learning. In order to successfully realize this core idea, we proposed a series of methods. First, we did not use word encoding technology, instead using character encoding technology that can retain more fine-grained information. Secondly, in order to supplement semantic information as much as possible and improve the dimension of feature vectors, interactive features were introduced. Next, we used the group strategy to transform the interactive feature matrix into several feature mapping groups, thus successfully transforming the traditional learning objects into feature mapping groups. Finally, we used MCNN-IFGS to perform feature learning on the feature map group. The experimental results show that our proposed MCNN-IFGS method was superior to the comparison method. In the paper, we used a two-dimensional convolution structure, which can also handle pictures well, which lays a good foundation for our future research on multimodal sentiment analysis. 

## Figures and Tables

**Table 1:** General statistics of Chinese review dataset.

Sentiment Polarity	Number		Avg_len
	Train	Test	
positive	36,450	12,150	32.58
negative	36,450	12,150	

**Table 2:** Distribution of text length in Chinese review dataset.

Length	10	20	30	40	50	>50
Number	30,910	22,973	13,820	8984	5492	17,821
Probability	0.31	0.23	0.14	0.09	0.05	0.18

**Table 3:** The results of different methods on Chinese review dataset. Here, each method was run five times. The value before “±” was the mean value, followed by the standard deviation.

Methods	Accuracy	Precision	Recall	F1
LSTM	0.932 ± 0.001	0.931 ± 0.002	0.934 ± 0.003	0.933 ± 0.001
2-layer LSTM	0.935 ± 0.001	0.932 ± 0.002	0.933 ± 0.002	0.932 ± 0.001
BiLSTM	0.931 ± 0.000	0.932 ± 0.004	0.932 ± 0.005	0.932 ± 0.000
2-layer BiLSTM	0.930 ± 0.001	0.932 ± 0.003	0.929 ± 0.003	0.931 ± 0.001
GRU	0.867 ± 0.002	0.871 ± 0.003	0.864 ± 0.006	0.868 ± 0.002
BiGRU	0.930 ± 0.001	0.928 ± 0.002	0.933 ± 0.003	0.931 ± 0.001
Character-level ConvNets	0.928 ± 0.000	0.929 ± 0.004	0.930 ± 0.006	0.929 ± 0.001
SLCABG	0.934 ± 0.000	0.931 ± 0.005	0.937 ± 0.006	0.934 ± 0.000
MDMLSM	0.930 ± 0.001	0.931 ± 0.002	0.929 ± 0.003	0.930 ± 0.001
MCNN-IFGS (word-based)	0.970 ± 0.004	0.978 ± 0.004	0.966 ± 0.006	0.972 ± 0.004
MCNN-IFGS (Ours)	**0.972 ± 0.003**	**0.974 ± 0.006**	**0.975 ± 0.008**	**0.974 ± 0.003**

**Table 4:** Performance of MCNN-IFGS on the Chinese review dataset. Here, different learning rates were used to carry out experiments. The value before “ ± ” was the mean value, followed by the standard deviation.

Learning Rate	Accuracy	Precision	Recall	F1
0.001	**0.972 ± 0.003**	**0.974 ± 0.006**	**0.975 ± 0.008**	**0.974 ± 0.003**
0.002	0.965 ± 0.006	0.971 ± 0.007	0.966 ± 0.009	0.968 ± 0.005
0.003	0.969 ± 0.003	0.971 ± 0.002	0.973 ± 0.005	0.972 ± 0.002
0.004	0.955 ± 0.025	0.961 ± 0.012	0.957 ± 0.040	0.959 ± 0.024
0.005	0.933 ± 0.029	0.923 ± 0.031	0.957 ± 0.024	0.940 ± 0.026
0.006	0.913 ± 0.066	0.923 ± 0.055	0.917 ± 0.071	0.920 ± 0.062
0.007	0.890 ± 0.053	0.911 ± 0.047	0.886 ± 0.060	0.898 ± 0.050
0.008	0.818 ± 0.162	0.848 ± 0.121	0.777 ± 0.251	0.934 ± 0.209

**Table 5:** MCNN-IFGS performance comparison of different dropout values on the Chinese review dataset. The value before “ ± ” was the mean value, followed by the standard deviation.

Dropout	Accuracy	Precision	Recall	F1
0.2	0.964 ± 0.002	0.962 ± 0.002	0.973 ± 0.004	0.968 ± 0.002
0.3	0.962 ± 0.002	0.967 ± 0.007	0.965 ± 0.011	0.966 ± 0.002
0.4	0.966 ± 0.002	0.963 ± 0.006	0.975 ± 0.005	0.969 ± 0.002
0.5	0.967 ± 0.004	0.971 ± 0.010	0.968 ± 0.007	0.970 ± 0.003
0.6	0.968 ± 0.005	0.971 ± 0.004	0.971 ± 0.013	0.971 ± 0.005
0.7	0.967 ± 0.002	0.969 ± 0.005	0.971 ± 0.007	0.970 ± 0.002
0.8	**0.972 ± 0.003**	**0.974 ± 0.006**	**0.975 ± 0.008**	**0.974 ± 0.003**

## Data Availability

The data presented in this study are available on request from the first author. The data are not publicly available due to privacy.
